# Undiagnosed hypertension and diabetes among the elderly in Amirkola, North of Iran

**DOI:** 10.22088/cjim.10.3.303

**Published:** 2019

**Authors:** Seyed Reza Seyed Reza, Mohammadali Bayani, Ali Zabihi, Moghaddeseh Shakerian, Tara Habibian, Ali Bijani

**Affiliations:** 1 Social Determinants of Health (SDH) Research Center, Health Research Institute, Babol University of Medical Sciences, Babol, Iran; 2 Nursing Care Research Center, Babol University of Medical Sciences, Babol, Iran; 3 Student Research Committee, Babol University of Medical Sciences, Babol, Iran

**Keywords:** Diabetes Mellitus, Hypertension, Elderly, Prevalence

## Abstract

**Background:**

As populations of elderly grow, the prevalence of type 2 diabetes and hypertension increases. These diseases can be asymptomatic for a long time and cause irreversible damages to organs. Therefore the aim of this study was to evaluate the prevalence of undiagnosed hypertension and diabetes among the elderly in Amirkola.

**Methods:**

This is a descriptive/analytical cross-sectional study and a part of the first phase of a cohort study on the health status of the elderly in Amirkola (a city in the North of Iran) which has been conducted on all people aged 60 and over since 2011. The demographic information was collected using a questionnaire, the hypertension was diagnosed by measuring blood pressure in lying down- position and the diabetes was diagnosed by measuring fasting blood glucose level.

**Results:**

This study was conducted on 1568 elderly participants including 703 (44.8%) females and 865 (55.2%) males. The overall prevalence of diabetes was 30.6% of which, 23.3% was diagnosed and 7.4% was undiagnosed. Nearly one-fourth (24.1%) of the participants with diabetes were unaware of their disease. Thirty-one percent of the people with undiagnosed diabetes also had undiagnosed hypertension. The overall prevalence of hypertension in this study was 62.8%, including 41.2% diagnosed and 21.5% undiagnosed hypertension.

**Conclusion:**

Regarding the results of this study and the high prevalence of undiagnosed diabetes and hypertension in elderly, improving the individual’s general awareness and screening in older adults for timely management are necessary.

Diabetes mellitus is a threatening public health problem in both the developed and the developing countries and one of the most common ongoing issues in the world (1). It is a chronic metabolic disease characterized by hyperglycemia and high glycosylated hemoglobin levels (2, 3). Diabetes is one of the most important causes of cardiovascular complications, one of the most common causes of end-stage renal disease (ESRD) in the developed countries and also a cause of blindness (4). It was estimated that diabetes affected 285 million of the population between 20-79 years of age (6.4% of the total population) in 2010; and it will affect 439 million people (7.7 percent of the world population) until 2030 (5). Nearly 50% of the diabetes in the world is undiagnosed and undiagnosed diabetes is associated with increased risk of death. In a study in Iran, 22% of the elderly had diabetes mellitus that among them 27.5% did not know who had the disease (6). Undiagnosed type 2 diabetes has significant consequences for public health and is associated with factors such as gender, age, race, marital status, obesity and hypertension (7).

Hypertension is one of the most prevalent chronic diseases among the elderly (8). It is the most important cardiovascular disease risk factor, in as much as in 2010 hypertension caused 2.043 million deaths and 24.6% of all deaths in China (9). 

By the year 2030, 27 million people will be added to the population of patients with hypertension (a 9.9 % increase since 2010). Up to age 45, the percentage of hypertension is higher in men than in women; between 45 to 64 years of age, this percentage is equal between men and women and above these ages hypertension is more common in women than in men (10). 

According to another research in Netherlands, there was a positive relation between diabetes and hypertension and factors such as age, family history, and history of cardiovascular diseases, alcohol consumption and diet (11). In a study in Iran, 42.7% of the adult population were hypertensive, wherein whom 46.2% of them were aware of their illness (12).

Diabetes and hypertension are known as two comorbid conditions in patients. Hypertension is observed in patients with diabetes 1.5 to 2 times more than in patients without diabetes. Since these two diseases are manageable health conditions (11) and considering their prevalent and dangerous complications, due to lack of any comprehensive population based study in elderly in Iran. The aim of this study was to investigate the prevalence of undiagnosed hypertension and diabetes among the elderly in Amirkola.

## Methods

This descriptive/analytical cross-sectional study is a part of the first phase of Amirkola Health and Ageing Project (AHAP) that has been conducted on all elderly people since 2011 (13). Amirkola is a small city in northern area of Iran, near the Caspian Sea. Inclusion criteria were as follows: age 60 and over and having completed data about hypertension and diabetes mellitus. Elderly people with incomplete data were excluded. In this study, hypertension was diagnosed by measuring the blood pressure using Omron M3 Intelligence upper arm blood pressure monitor in lying down position twice (two different times) with the standard method (10). A systolic blood pressure greater or equal to 140 mmHg and or a diastolic blood pressure greater or equal to 90 mmHg were defined as hypertension. Moreover, diabetes was diagnosed based on WHO criteria and by fasting blood glucose level twice greater than or equal to 126 mg/dl (14). In this study, diagnosed hypertension or diabetes mellitus were defined by an answer of “yes” to this question, “Have you ever been told by a doctor that you have hypertension or diabetes?” Among those who answered “no,” undiagnosed hypertension and diabetes were defined by measuring blood pressure and fasting blood glucose as mentioned above.

Furthermore, fasting blood samples were taken from the elderly to measure the blood glucose and lipid and performing other biochemical tests. The participants’ weight was measured by minimal clothing using Seca digital weighing scale with an accuracy of 0.1 kg and their height was measured by audiometer with a possible error of 0.5 cm. The BMI was then calculated by dividing the weight in kilogram by the square of the height in meter. The demographic information including age, gender, level of education, smoking and marital status was collected through a questionnaire.

The collected data was then analyzed using SPSS 18.We used ANOVA to compare the mean of quantitative variables in three groups of hypertension and diabetes and chi-square test for comparison of prevalence of hypertension and diabetes by sex. After calculating the total prevalence of diabetes and hypertension and deducting the diagnosed (self-reported) cases from it, the prevalence of undiagnosed diabetes and hypertension was determined and then their relation with the demographic information and the risk factors were investigated. A p-value of less than or equal to 0.05 was considered to be statistically significant.

## Results

Among the 1616 investigated elderly participants, 1568 participants including 703 (44.8%) men and 865 (55.2%) women provided the required information were included in the study (table 1). In this study, 1087 (69.3%) participants were without diabetes while 481 (30.6%) were diabetic. The number of diagnosed diabetes was 365 (23.3%) while the undiagnosed diabetes was 116 (7.4%) (nearly one-fourth (24.1%) of the patients with diabetes were unaware of their disease). The prevalence of diagnosed diabetes was higher in women while the prevalence of undiagnosed diabetes was higher in men (p<0.001). Table 2 shows that fasting blood sugar, cholesterol, triglyceride, LDL and BMI were higher in participants with undiagnosed diabetes than in those with diagnosed diabetes or those without diabetes.

**Table1:** Frequency distribution and percentage of demographic characteristics of the elderly in Amirkola

**Investigated variables**		**Number (percentage)**
Marital status	Married	1341 (85.5)
	Widow	154 (9.8)
	Divorced	4 (0.3)
	Widower	69 (4.4)
Level of Education	Illiterate	1010 (64.4)
	Elementary School	424 (27.0)
	Middle school, High School, High School Diploma	89 (5.7)
	Associate’s Degree, Bachelor’s Degree, Master’s Degree	45 (2.9)
Employment status	Unemployed	98 (6.3)
	Housewife	625 (39.9)
	Retired	345 (22.0)
	Employed (Housewives Excluded)	491 (31.3)
	Unspecified	9 (6.0)
Age	60-64	556 (35.5)
	65-69	325 (20.7)
	70-74	274 (17.5)
	75-79	246 (15.7)
	80-84	113 (7.2)
	85≥	54 (3.4)
Smoking	No	1277 (81.4)
	Yes	291 (18.6)

**Table 2:** Mean and standard deviation of risk factors for diabetes among the elderly in Amirkola

	**Without diabetes** **Mean±SD**	**Diagnosed diabetes** **Mean±SD**	**Undiagnosed diabetes** **Mean±SD**	**P-value**
FBS	98 11.5	161.7±63.1	166.3±48.7	0.000
Cholesterol	196.7±40	189.9±46.1	207.1±49.9	0.000
Triglyceride	151±76.2	178.7±97.3	190.7±93.7	0.000
LDL	131±41.8	119.5±44.2	140.7±57.2	0.000
HDL	38.6±4.7	38.6±4.7	39± 4.45	0.591
Age	69.7±7.5	68.2±6.7	69.8±7.8	0.oo4
BMI	26.7±4.5	28±4.5	28.4±4.4	0.000

In this study, prevalence of diabetes (diagnosed and undiagnosed) in elderly had a significant relation with employment status, age and BMI and in fact the highest prevalence was among housewives and 60 to 64 age range group. The participants with undiagnosed diabetes had the highest rates of overweight and obesity (BMI ≥ 25). Moreover, diabetes had no significant relationship with marital status, level of education and smoking. Thirty-one percent of the participants with undiagnosed diabetes also had undiagnosed hypertension. In this study, 582 (37.1%) participants did not have hypertension and 986 (62.8%) participants had hypertension. The number of diagnosed hypertension was 648 (41.3%) and the number of undiagnosed hypertension was 338 (21.6%) (More than one-third (34.2%) of the participants with hypertension were not aware of their disease). The prevalence of diagnosed hypertension was higher in women and prevalence of undiagnosed hypertension was higher in men, but overall, hypertension was more prevalent in women (68%) than in men (58.8%) (p<0.001). Table 3 shows people with undiagnosed hypertension were older and had higher total and LDL cholesterol levels compared to those with diagnosed hypertension.

**Table 3:** Mean and standard deviation of risk factors for hypertension among the elderly in Amirkola

	**Without hypertension** **Mean±SD**	**Diagnosed hypertension** **Mean±SD**	**Undiagnosed hypertension** **Mean±SD**	**P-value**
FBS	113.3±42.6	122.3±46.8	117.4±47.7	0.003
Cholesterol	197.8±42.9	193.1±42.3	197.7±41.9	0.105
Triglyceride	153.9±84.0	164.8±81.5	163.1±88.6	0.062
LDL	132.1±44.7	125.5±42.4	130.9±45.7	0.022
HDL	38.6±4.3	38.5±4.3	38.9±4.3	0.391
Age	68.1±7.3	70.0±7.3	70.3±7.49	0.000
BMI	26.1±4.3	28.1±4.6	27.0±4.3	0.000

The prevalence of hypertension in elderly had a significant relation with marital status, employment status, age, smoking and a high BMI. The highest rates of undiagnosed hypertension were observed in married people (86.1%) and employed ones other than housewives (39.3%). About 65.7% of participants with undiagnosed hypertension had a BMI greater than or equal to 25 (were obese or overweight). In the present study, hypertension had no relation with the level of education. Moreover, 10.7% of people with undiagnosed hypertension also had undiagnosed diabetes.

## Discussion

In this study, the total prevalence of diabetes among the elderly people in Amirkola was 30.6% the prevalence of diabetes was 34.2% in India (11), 27.4% (among people over 65) in Tunisia (15), 38.3% (among people over 50) in Pakistan (16) and 21.2% (among people over 65) in United States (17). These studies indicate that the prevalence of diabetes in the developing countries such as Tunisia, Pakistan, India and Iran is higher than in the developed countries. This can be due to rapid changes in lifestyle caused by urbanization which itself resulted from migration of people from rural to urban areas in the developing countries (18). There are various factors that can explain the high prevalence of diabetes mellitus among the elderly. One can be the fact that they are more prone to insulin deficiency and insulin resistance, especially the obese ones. Moreover, there are other factors including decreased physical activities, stress, illness and medications that can accelerate the onset of this disease (19). 

The prevalence of undiagnosed diabetes in our entire study population was 7.4%. Although health care facilities are widely accessible in our country, nearly one-fourth (24.1%) of the participants with diabetes (116 out of 481 participants) were not aware of their diseases. In a study in the United States, about 8% of the participants were diabetic, that from whom nearly 25% were not aware of their diseases (20). In Malaysia (55%) (19) and Malawi (41%) (21) of people with diabetes were not aware of their diseases. Although health education programs and announcements have increased among our population, the results show that these activities have not been enough to reduce the percentage of undiagnosed diabetes cases by themselves; and also indicate the urgent requirement for a serious screening program for this vulnerable population.

In the present study, the prevalence of diabetes was higher among women (36.1%) than among men (26.3%), but the prevalence of undiagnosed diabetes was higher in men (53.4%). In another study in China, the prevalence of diabetes among people older than 60 was more common in women than in men (9). In Germany, the prevalence of diabetes did not differ between men and women (22). The higher prevalence of diabetes in women compared to men might be due to the fact that women spend most of their time at home and as a result have less physical activities. Moreover, since women go to medical centers more often than men, they visit the doctors more providing a greater chance for the doctors to examine, diagnose and cure their undiagnosed diabetes.

In the present research, diabetes was significantly associated with factors including cholesterol, BMI, LDL, triglyceride, hypertension and age. The results also showed that there was no relation between HDL and diabetes. In addition, diabetes was not associated with marital status, level of education and smoking, but it had a significant relation with employment status and its highest prevalence was among housewives (36%). In another study, diabetes was significantly related to total cholesterol, triglyceride, LDL and had no significant relation with HDL (23). Moreover, in a study in turkey, prevalence of diabetes was associated with age, BMI, a low level of education, living alone and smoking (24). 

In the present study, the total prevalence of hypertension among the elderly in Amirkola was 62.8%. In several other studies, this rate was as follows: 68% among urban elderly in Brazil (25), 61.7% among people aged 45 to 64 years in Norway (26), 62.6% in Malaysia (19), 70.8% among people older than 65 in the United States (17). Having considered the high prevalence of hypertension in elderly and the importance of the risks it causes, it is necessary that health team staffs and community health nurses implement proper interventions to prevent hypertension and its complications.

The prevalence of undiagnosed hypertension among our whole- study population was 21.6%. Although healthcare facilities are widely accessible in our country, 338 out of 986 participants (34.2%) with hypertension in the present study, or in other words, one-third of the participants with hypertension were not aware of their diseases. In Norway (23.1%) (26), in Malawi (58%) (21) and in Malaysia more than one-third (35%) (19) of the people with hypertension were not aware of their disease. Therefore, screening and effective treatment of hypertension should be considered as an important goal by healthcare experts and community health nurses. 

In the present study, the prevalence of hypertension was higher among women (68%) than men (58.8%); however, the prevalence of undiagnosed hypertension was higher among men (63.9%) and women were more aware of their diseases. In a study in Tanzania on people older than 70, hypertension was more common among women than among men (27) and in a study in the United States, like our study, the prevalence of hypertension was higher among women (76.6%) than among men (63%) (17). The results of these studies indicate that probably women are more inclined to know and measure their blood pressure and we need to take more measures to detect hypertension in men. 

In the present study, hypertension was significantly associated with age, marital status, employment status and smoking, but it was not associated with the level of education. Undiagnosed hypertension was more prevalent among married people (86.1%) and employed people other than housewives (39.3%) compared to other groups. Furthermore, in the present study, hypertension was significantly related to factors including FBS, BMI and LDL, but it had no relationship with cholesterol, triglyceride and HDL. In another study in North of Jordan, elderly people with hypertension had high level of FBS, LDL, TG (triglyceride), TC (total cholesterol), and low level of HDL than those without hypertension and there was no association with BMI (28). The present study was conducted as a cohort study with a high participation rate of the elderly in Amirkola (1616 out of 2234 potential participants), which can be regarded as its strength. On the other hand, to point out some of the limitations, it is worth mentioning that we disregarded some factors associated with diabetes and hypertension such as family history, health record, economic and social status, diet and physical activities. Apart from it, the fact that the diseases investigated by the present researchers are self-reported; and would deprive us of a more accurate prevalence estimate of these diseases in elderly and to some extent affects the results.

In conclusion, the present study indicates the high prevalence of undiagnosed diabetes and hypertension in elderly and reflects the necessity of a serious and accurate screening program for this vulnerable population. Different environmental factors and lifestyle play an important role in the incidence of these diseases. Therefore, considering the growing population of elderly and their substantial illiteracy, preventive interventions for these diseases should be prioritized and appropriate programs should be planned to reach the goal of having healthy elderly in the society. In addition, making decisions to adopt a healthy lifestyle, especially in the last years of life should be considered as an important issue. 
